# Evaluation of Unreimbursed Medicaid Costs Among Nonprofit and For-Profit US Hospitals

**DOI:** 10.1001/jamanetworkopen.2021.48232

**Published:** 2022-02-14

**Authors:** Ge Bai, Hossein Zare, David A. Hyman

**Affiliations:** 1 Johns Hopkins Carey Business School, Johns Hopkins University, Baltimore, Maryland; 2 Johns Hopkins Bloomberg School of Public Health, Johns Hopkins University, Baltimore, Maryland; 3 Global Health Services and Administration, University of Maryland Global Campus, Adelphi; 4 Georgetown University Law Center, Washington, DC

## Abstract

This economic evaluation uses 2019 Medicare cost report data to examine the unreimbursed Medicaid costs among nonprofit and for-profit US hospitals.

## Introduction

Nonprofit US hospitals receive sizeable subsidies, including exemption from federal and state taxes and the ability to issue tax-free bonds. An estimate from 2015 placed the value of these subsidies at almost $25 billion.^1^ Nonprofit hospitals are required to provide various forms of community benefit to justify their tax-related subsidies, although federal law does not impose a minimum requirement.^2^ Among the 8 components of community benefit reported by nonprofit hospitals on Internal Revenue Service Form 990, the largest is unreimbursed Medicaid costs (44%).^3,4^ We used 2019 Medicare cost report data to examine unreimbursed Medicaid costs among nonprofit and for-profit US hospitals.

## Methods

This economic evaluation was deemed exempt from review by the Johns Hopkins University Institutional Review Board, because no identifiable private data or identifiable biospecimens were accessed. The study followed the Consolidated Health Economic Evaluation Reporting Standards (CHEERS) reporting guideline.

We used the 2019 Medicare cost reports to obtain information on self-reported unreimbursed Medicaid costs. Our sample included 3446 private hospitals (2617 nonprofit and 829 for-profit hospitals). Unreimbursed Medicaid costs were obtained from Worksheet S-10, calculated as the estimated cost for treating Medicaid patients (ie, the charges for the services multiplied by the hospital’s cost to charge ratio) minus all reimbursements and supplemental payments.^5^

For each state, we calculated (1) the weighted mean unreimbursed Medicaid cost to expense ratio (the total unreimbursed Medicaid cost across hospitals divided by their total expenses) for all private hospitals, nonprofit hospitals, and for-profit hospitals and (2) the ratio of the weighted mean unreimbursed Medicaid cost to expense ratio of for-profit hospitals relative to nonprofit hospitals (ie, the for-profit to nonprofit relative ratio). This weighted mean approach is consistent with the prior literature on hospital charity care.^6^ We also examined the median for-profit to nonprofit relative ratios for states that did (and did not) expand Medicaid by January 1, 2019.

The median test was used to compare medians in unreimbursed Medicaid costs of nonprofit and for-profit hospitals. Statistical significance was set at *P* < .01 (2-sided). Statistical analysis was conducted using Stata, version 15 (StataCorp).

## Results

In 2019, the 3446 US private hospitals in our data set incurred $20.59 billion in unreimbursed Medicaid costs, representing 2.52% of their total expenses. Nevada hospitals had the highest weighted mean unreimbursed Medicaid cost to expense ratio (7.74%), whereas hospitals in Maryland (0.02%), Utah (0.07%), and Alabama (0.14%) had the lowest ratios (Table). In 23 of the 45 states (51.1%) in which both nonprofit and for-profit hospitals operated, for-profit hospitals had higher unreimbursed Medicaid cost to expense ratios than nonprofit hospitals (Figure).

**Table.** Weighted Mean Unreimbursed Medicaid Costs in 2019 by State

State	Implemented Medicaid expansion	Total unreimbursed Medicaid cost, $ (millions)	Hospital weighted mean unreimbursed Medicaid cost to expense ratio, %			For-profit to nonprofit relative ratio^a^
			Private	Nonprofit	For-profit	
Alaska^b^	Yes	4.99	0.29	0.23	0.47	2.00
Alabama^b^	No	7.27	0.14	0.14	0.14	1.00
Arkansas^b^	Yes	80.00	1.32	1.28	1.51	1.18
Arizona	Yes	766.50	4.77	4.80	4.61	0.96
California^b^	Yes	3391.00	3.74	3.73	3.86	1.04
Colorado^b^	Yes	528.00	4.46	4.29	5.30	1.23
Connecticut^b^	Yes	537.90	4.56	4.54	5.09	1.12
District of Columbia	Yes	8.04	0.23	0.27	0.00	0.00
Delaware	Yes	32.30	0.95	0.95	NA	NA
Florida^b^	No	1687.00	3.92	3.16	5.62	1.78
Georgia	No	162.90	0.92	0.98	0.55	0.56
Hawaii	Yes	54.20	1.72	1.72	NA	NA
Iowa^b^	Yes	77.51	1.34	1.30	4.53	3.49
Idaho	No	50.66	1.09	1.19	0.80	0.67
Illinois^b^	Yes	947.70	2.81	2.79	3.24	1.16
Indiana	Yes	546.00	2.60	2.75	1.21	0.44
Kansas	No	40.23	0.71	0.88	0.36	0.41
Kentucky	Yes	99.59	0.89	0.91	0.67	0.73
Louisiana^b^	Yes	160.10	1.40	1.38	1.51	1.10
Massachusetts^b^	Yes	530.00	1.88	1.86	1.94	1.05
Maryland	Yes	2.78	0.02	0.02	NA	NA
Maine	Yes	82.60	1.40	1.40	NA	NA
Michigan	Yes	562.20	1.95	1.99	1.17	0.59
Minnesota	Yes	367.00	2.32	2.32	NA	NA
Missouri^b^	No	169.50	0.88	0.81	1.70	2.09
Mississippi^b^	No	12.43	0.28	0.16	0.70	4.45
Montana^b^	Yes	12.52	0.28	0.23	0.96	4.28
North Carolina^b^	No	93.20	0.48	0.46	0.68	1.49
North Dakota^b^	Yes	41.60	0.99	0.72	3.62	5.06
Nebraska	No	115.48	1.81	1.87	0.90	0.48
New Hampshire	Yes	170.85	3.06	3.10	2.36	0.76
New Jersey^b^	Yes	591.90	2.46	2.27	4.65	2.04
New Mexico	Yes	52.29	1.20	1.46	0.57	0.39
Nevada^b^	Yes	429.00	7.74	7.66	7.79	1.02
New York	Yes	2220.00	3.66	3.70	0.00	0.00
Ohio	Yes	1608.40	4.36	4.39	3.26	0.74
Oklahoma^b^	No	30.82	0.32	0.14	0.72	5.04
Oregon	Yes	535.34	4.73	4.83	1.34	0.28
Pennsylvania	Yes	949.70	2.08	2.11	1.71	0.81
Rhode Island^b^	Yes	116.70	3.04	2.73	6.33	2.32
South Carolina	No	43.37	0.52	0.63	0.19	0.30
South Dakota	No	56.19	1.21	1.28	0.13	0.10
Tennessee^b^	No	323.90	1.86	1.78	2.13	1.19
Texas^b^	No	325.90	0.69	0.13	1.36	10.51
Utah	No	3.89	0.07	0.09	0.02	0.27
Virginia	Yes	201.50	1.10	1.19	0.64	0.54
Vermont	Yes	117.00	4.25	4.25	NA	NA
Washington	Yes	620.14	3.81	3.86	0.80	0.21
Wisconsin^b^	No	663.20	3.16	3.13	4.13	1.32
West Virginia	Yes	339.80	4.78	4.92	3.19	0.65
Wyoming	No	14.73	2.00	2.10	1.57	0.75
United States^c^	NA	20 585.82	2.52	2.51	2.53	1.00
State median (IQR)	NA	NA	1.72 (0.88-3.16)	1.72 (0.81-3.13)	1.36 (0.67-3.26)	1.00 (0.54-1.49)

Abbreviation: NA, not applicable.

^a^ The for-profit to nonprofit relative ratio is calculated as the weighted mean unreimbursed Medicaid cost to expense ratio for for-profit hospitals divided by the ratio for nonprofit hospitals in each state.

^b^ States with for-profit to nonprofit relative ratios greater than 1.

^c^ Weighted mean values are at the national level.

**Figure.**
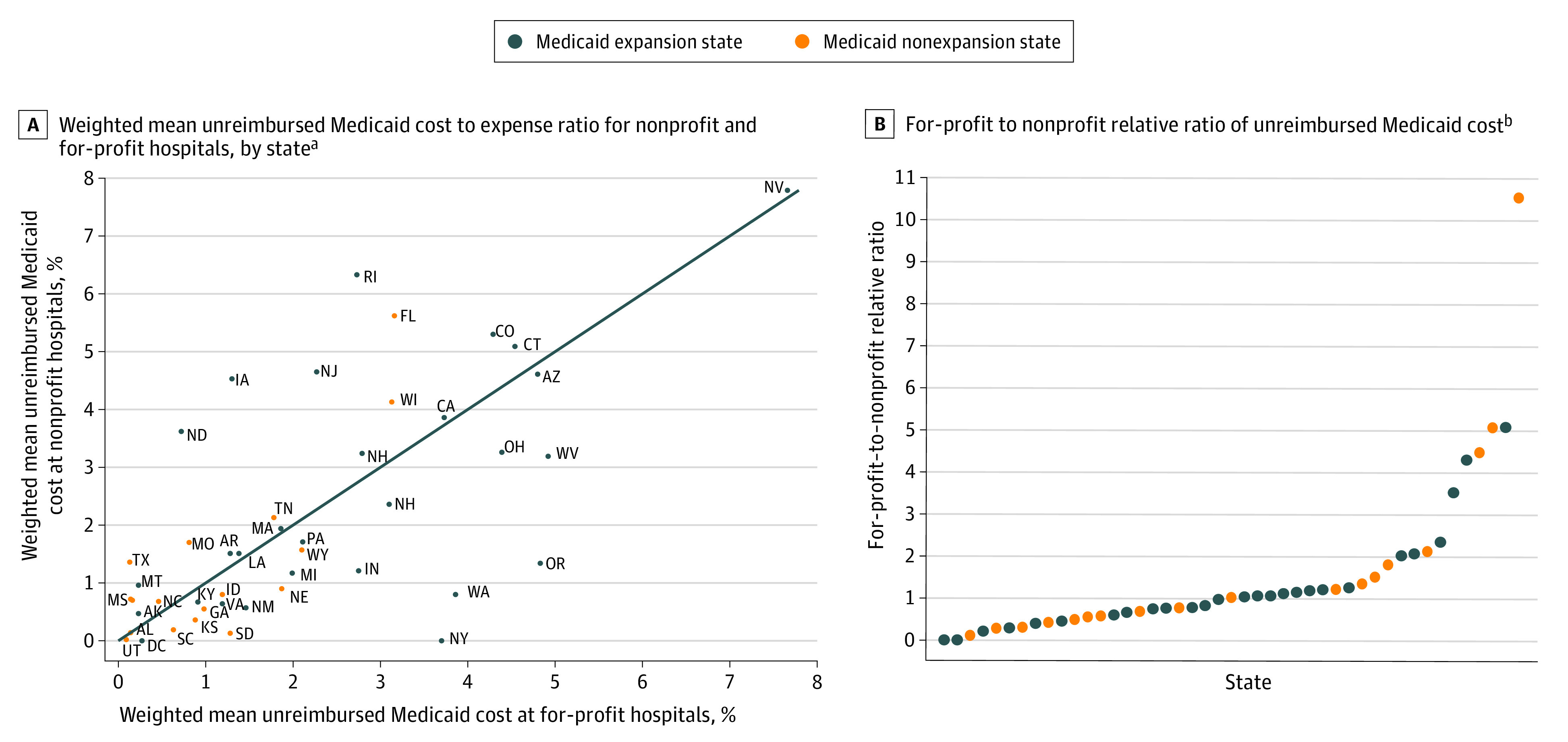
Hospital Unreimbursed Medicaid Costs in 2019, by Ownership Type and State A, Weighted mean unreimbursed Medicaid cost to expense ratio (%) for nonprofit hospitals and for-profit hospitals. B, For-profit to nonprofit relative ratio of unreimbursed Medicaid cost. ^a^States that had implemented the Medicaid expansion by January 1, 2019, are indicated in blue; other states are indicated in orange. ^b^The for-profit to nonprofit relative ratio is calculated as the weighted mean unreimbursed Medicaid cost to expense ratio for for-profit hospitals divided by the ratio for nonprofit hospitals in each state. States that had implemented the Medicaid expansion by January 1, 2019, are indicated in blue; other states are indicated in orange. The 45 states that have both nonprofit and for-profit hospitals were ranked by their for-profit to nonprofit relative ratios.

Texas had the highest ratio of for-profit to nonprofit unreimbursed Medicaid costs to expenses (10.51:1), whereas New York and the District of Columbia had the lowest ratio (0.00:1). Of the 45 states in which both nonprofit and for-profit hospitals operated, the 28 states that implemented the Medicaid expansion had a median (IQR) for-profit to nonprofit relative ratio that was similar to the 17 states that did not expand Medicaid (0.99 [0.56-1.21] vs 1.00 [0.48-1.77]; *P* = .85).

## Discussion

In this economic evaluation, nonprofit and for-profit hospitals had similar unreimbursed Medicaid costs as a share of expenses. In half of the 45 states in which both nonprofit and for-profit hospitals operate, nonprofit hospitals had a lower weighted mean unreimbursed Medicaid cost to expense ratio than for-profit hospitals—but only nonprofit hospitals receive a sizeable tax subsidy. Thus, our results suggest that the largest component of community benefit supposedly provided by nonprofit hospitals (ie, unreimbursed Medicaid costs, net of supplemental payments) is poorly aligned with the (effectively automatic) tax subsidy that these institutions receive. Prior research suggested similar results for the provision of charity care by nonprofit vs for-profit hospitals.^6^

In part, supplemental payments are designed to help offset unreimbursed Medicaid costs. A sizeable majority of hospitals in our data set (2375 [69%]) reported receiving supplemental payments, including 1840 nonprofit hospitals and 535 for-profit hospitals. The Medicare cost reports do not contain sufficient information for us to analyze the distribution of unreimbursed Medicaid costs if there were no supplemental payments, which is a limitation of this study. Our analysis also assumes that the figures reported by hospitals on their Medicare cost reports (under penalty of law) are accurate. If hospitals erroneously underreport supplemental payments, then their unreimbursed Medicaid costs will be inflated.

Policy makers should do more to address these issues, including providing greater transparency about the magnitude of the subsidies received by nonprofit hospitals and establishing a more direct nexus between these subsidies and the performance of those facilities in providing community benefit, whether in the form of unreimbursed Medicaid costs, charity care, or some other measure.
